# Serum Aberrant *N*-Glycan Profile as a Marker Associated with Early Antibody-Mediated Rejection in Patients Receiving a Living Donor Kidney Transplant

**DOI:** 10.3390/ijms18081731

**Published:** 2017-08-08

**Authors:** Daisuke Noro, Tohru Yoneyama, Shingo Hatakeyama, Yuki Tobisawa, Kazuyuki Mori, Yasuhiro Hashimoto, Takuya Koie, Masakazu Tanaka, Shin-Ichiro Nishimura, Hideo Sasaki, Mitsuru Saito, Hiroshi Harada, Tatsuya Chikaraishi, Hideki Ishida, Kazunari Tanabe, Shigeru Satoh, Chikara Ohyama

**Affiliations:** 1 Department of Urology, Hirosaki University Graduate School of Medicine, Hirosaki 036-8562, Japan; noro_daisuke@yahoo.co.jp (D.N.); shingoh@hirosaki-u.ac.jp (S.H.); tobisawa@hirosaki-u.ac.jp (Y.T.); moribio@hirosaki-u.ac.jp (K.M.); goodwin@hirosaki-u.ac.jp (T.K.); coyama@hirosaki-u.ac.jp (C.O.); 2 Department of Advanced Transplant and Regenerative Medicine, Hirosaki University Graduate School of Medicine, Hirosaki 036-8562, Japan; bikkuri@opal.plala.or.jp; 3 Graduate School of Life Science, Frontier Research Center for Advanced Material and Life Science, Hokkaido University, Sapporo 060-0810, Japan; tanaka@soyaku.co.jp (M.T.); shin@sci.hokudai.ac.jp (S.-I.N.); 4 Department of Urology, St. Marianna University of Medicine, Kawasaki 216-8511, Japan; sr20det@marianna-u.ac.jp (H.S.); t.chikaraishi@gmail.com (T.C.); 5 Department of Urology, Akita University Graduate School of Medicine, Akita 010-8543, Japan; urosaito@gmail.com (M.S.); shigerus@doc.med.akita-u.ac.jp (S.S.); 6 Department of Kidney Transplant Surgery, Sapporo City General Hospital, Sapporo 060-8611, Japan; hirohara80@mac.com; 7 Department of Urology, Tokyo-Woman’s Medical University, Tokyo 162-8666, Japan; tgphide@gol.com (H.I.); k-tanabe@k3.dion.ne.jp (K.T.)

**Keywords:** biomarker, antibody-mediated rejection, *N*-glycan

## Abstract

We determined if the serum *N*-glycan profile can be used as a diagnostic marker of antibody-mediated rejection (ABMR) in living donor kidney transplant (LKTx) recipients. Glycoblotting, combined with mass spectrometry, was used to retrospectively examine *N*-glycan levels in the postoperative sera of 197 LKTx recipients of whom 16 recipients had ABMR with or without T-cell-mediated rejection (TCMR), 40 recipients had TCMR, and 141 recipients had no adverse events. Multivariate discriminant analysis for prediction of ABMR was performed by inputting an ABMR event as an explanatory variable and sex, age, and serum *N*-glycan level as objective variables. The *N*-glycan score was calculated by multiplying the level of candidate objective variables by objective function values. The ABMR predictive performance of the *N*-glycan score was assessed by receiver operator characteristic curve and Kaplan–Meier curve analyses. The *N*-glycan score discriminated ABMR with 81.25% sensitivity, 87.85% specificity, and an area under the curve (AUC) of 0.892 that was far superior to that of preformed donor-specific antibody status (AUC, 0.761). Recipients with *N*-glycan-positive scores >0.8770 had significantly shorter ABMR survival than that of recipients with *N*-glycan-negative scores. Although the limitations of our study includ its small sample size and retrospective nature, the serum *N*-glycan score may contribute to prediction of ABMR.

## 1. Introduction

Antibody-mediated rejection (AMBR) is a widely recognized cause of allograft loss in kidney transplant recipients. In the last decade, although the ABMR rate has been significantly reduced, ABMR has remained a common complication that can affect long-term graft survival [1]. In recent years, the role of donor-specific anti-HLA antibodies (DSAs) in ABMR has been characterized thoroughly, and this concept has been developed into a DSA detection system utilizing Luminex^®^ assays to improve risk stratification for allograft loss. The affinity of circulating DSAs [2] and their ability to bind complements [3,4,5] have been implicated in poor allograft outcomes. Recently, evaluation of the DSA immunoglobulin IgG subclass identified distinct phenotypes of kidney allograft ABMR [6]. Although the recent evolution of DSA detection methods may be a step forward in the development of risk stratification models of ABMR, the use of DSA, complement, or DSA IgG subtyping for prediction of ABMR has not been incorporated into routine clinical practice yet. Thus, there is a need for novel serum markers to improve diagnostic accuracy and predict longer graft survival after a transplant. Recently, Malard-Castagnet et al. reported that higher levels of sialylated IgG were detectable on the day of the transplant in patients who did not develop ABMR; they had higher levels of sialylated class I DSA at the initial detection of DSA. This was the first report suggesting that transplant outcome, and particularly ABMR, is associated with levels of sialylated IgG antibodies [7]. This research suggested that immunoreaction-associated aberrant glycosylation of a serum glycoprotein could be used as a serum-based predictive biomarker of ABMR.

Glycosylation has an important role in various biological functions. Recently, our group demonstrated that high-throughput, comprehensive, and quantitative serum *N*-glycomics was a promising method to screen *N*-glycan for diagnostic and prognostic markers of several cancers [8,9,10] and was a promising predictive tool for patients undergoing hemodialysis [11]. However, the use of serum *N*-glycans as a predictive biomarker of ABMR has not yet been tested. In the present study, we performed serum *N*-glycomics in transplant patients and evaluated its potential as a predictive serum-based biomarker of early ABMR.

## 2. Results

### 2.1. The Level of Serum Sialyl Hybrid Type and Sialyl Bisecting Type N-Glycans Were Associated with Recipients Who Developed ABMR and Much Lower than That of Recipients Who Did Not Develop ABMR

**Figure 1:**
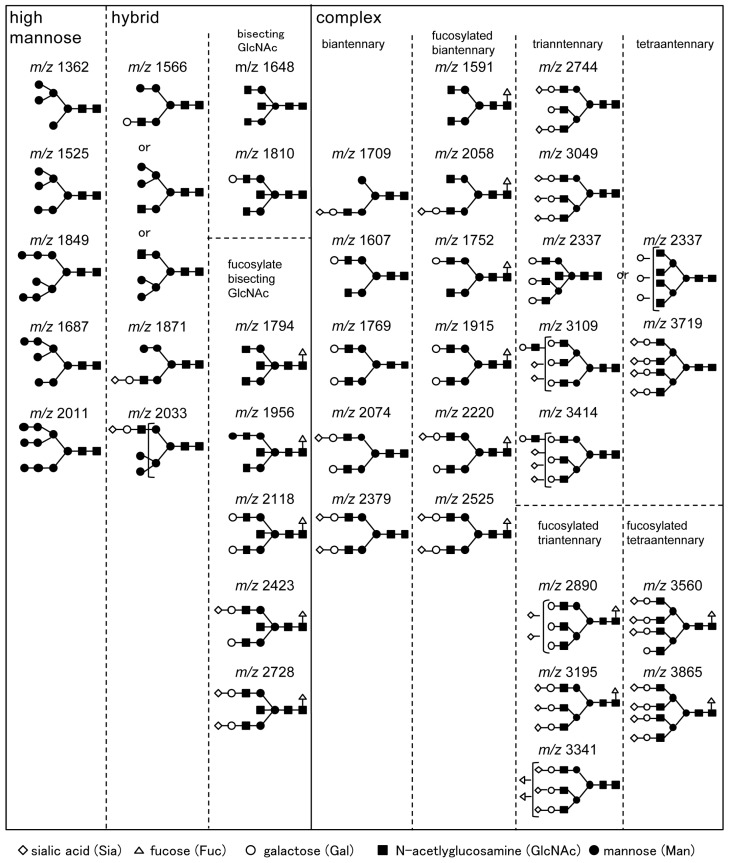
Schematic representation of 36 types of *N*-glycans identified by *N*-glycomics. Putative structures of *N*-glycans are presented by means of monosaccharide symbols. Clear circles, galactose (Gal); black circles, mannose (Man); black squares, *N*-acetylglucosamine (GlcNAc); clear triangles, fucose (Fuc); and clear diamonds, *N*-acetylneuraminic acid (sialic acid). *m*/*z* represent mass divided by charge number and horizontal axis in a mass spectrum is expressed in units of *m*/*z*.

Serum *N*-glycomics identified 36 types of *N*-glycans (Table 1, Figure 1) that had good quantitative reproducibility among all samples and could be used for statistical analysis.

**Figure 2:**
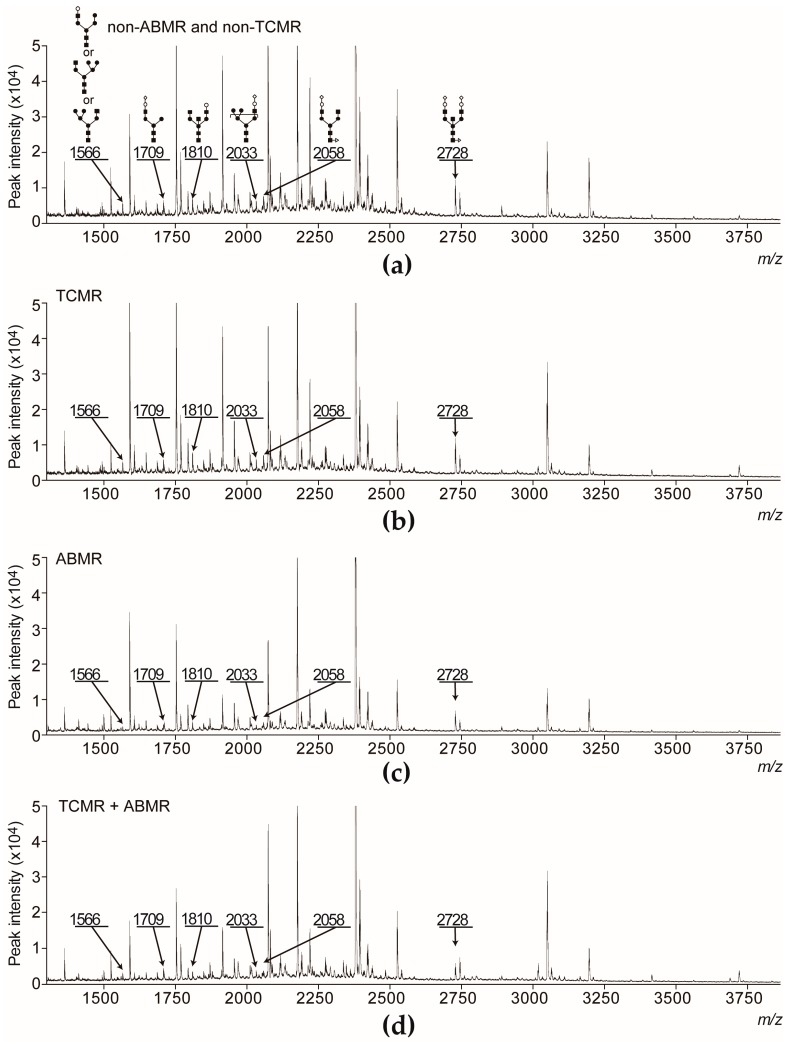
Representative MALDI-TOF mass spectra (*m*/*z* range of 1250 to 3865) of benzyloxyamine (BOA)-labeled *N*-glycans derived from the serum of patients one day before LKTx without ABMR (non-ABMR; no adverse events and TCMR alone) and with ABMR (with or without TCMR). (**a**) A mass spectrum of serum *N*-glycans from a patient who did not develop any adverse events; (**b**) A mass spectrum of serum *N*-glycans from a patient who developed TCMR; (**c**) A mass spectrum of serum *N*-glycans from a patient who developed ABMR; (**d**) A mass spectrum of serum *N*-glycans from a patient who developed ABMR and TCMR. The peaks of ABMR-related *N*-glycans (*m*/*z* 1566, 1709, 1810, 2033, 2058, and 2728) are indicated in the mass spectra. The putative structures of *m*/*z* 1566, 1709, 1810, 2033, 2058, and 2728 are indicated by monosaccharide symbols.

The characteristics of the healthy volunteers (HLT) and of the patients in the non-ABMR and ABMR groups are shown in Table 2. There were no statistically significant differences in age, sex, and ABO-incompatibility status between non-ABMR and ABMR group. The preformed-DSA-positive status and non-ABMR and non-TCMR survival periods were significantly different between the two groups. The majority of ABMR cases developed ≤1 month after LKTx. Representative MALDI-TOF mass spectra are shown in Figure 2a–d.

**Figure 3:**
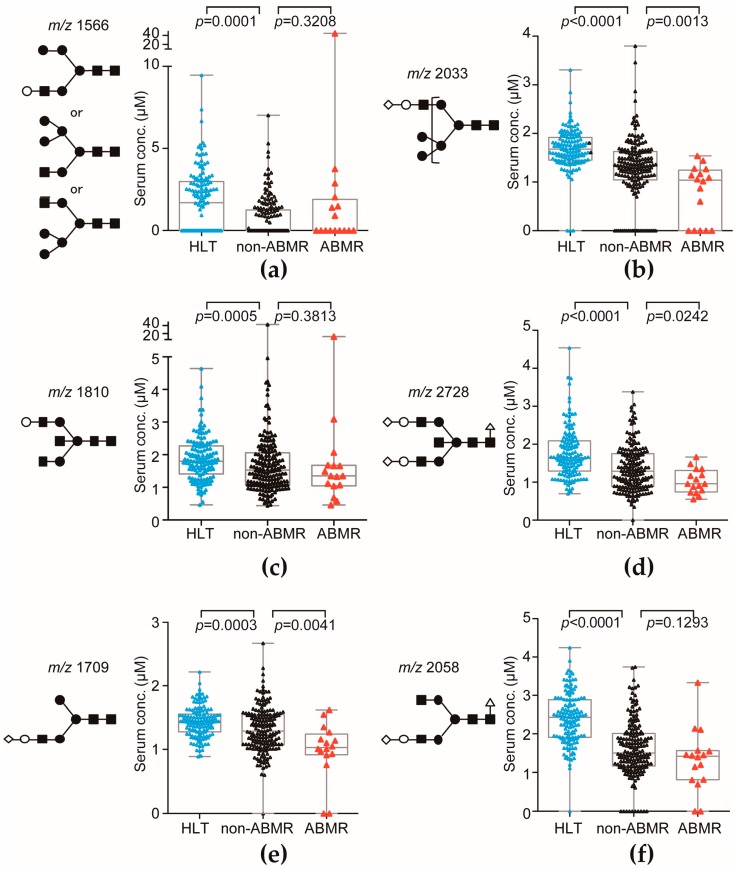
The levels of ABMR-associated *N*-glycans in the healthy volunteers (HLT) and recipients on postoperative day 1. (**a**) Serum *m*/*z* 1566 levels in the HLT (cian triangle), non-ABMR (black triangle), and ABMR (red triangle) patients; (**b**) Serum *m*/*z* 2033 levels in the HLT, non-ABMR, and ABMR patients; (**c**) Serum *m*/*z* 1810 levels in the HLT, non-ABMR, and ABMR patients; (**d**) Serum *m*/*z* 2728 levels in the HLT, non-ABMR, and ABMR patients; (**e**) Serum *m*/*z* 1709 levels in the HLT, non-ABMR, and ABMR patients; (**f**) Serum *m*/*z* 2058 levels in the HLT, non-ABMR, and ABMR patients. Putative structures of *N*-glycans are indicated by monosaccharide symbols.

To identify the ABMR-related aberrant *N*-glycosylation in serum, we performed multivariate discriminant analysis by inputting ABMR event as an explanatory variable and recipient’s sex, age, and serum *N*-glycan level as objective variables. We found six *N*-glycans (hybrid type: *m*/*z* 1566, 2033; bisecting type: *m*/*z* 1810, 2728; complex biantennary type: *m*/*z* 1709, 2058) related to detection of ABMR that had calculated *F* values > 2.0 by multivariate discriminant analysis (Figure 3 and Table 3). Of those three ABMR-related terminal sialylated *N*-glycans (hybrid type: *m*/*z* 2033; bisecting type: *m*/*z* 2728; complex biantennary type: *m*/*z* 1709), the serum levels were significantly lower in the recipients who developed ABMR than in those who did not (Figure 3). ABMR-related terminal sialylated *N*-glycans level of HLT was significantly higher than non-ABMR and ABMR groups. To discriminate recipients who developed ABMR by using the whole aberrant *N*-glycosylation profile, the ABMR diagnostic *N*-glycan scores were calculated according to the following formula:

*N*-glycan score = (*Age* × −0.0024) + (*Sex* × −0.9142) + (*m*/*z* 1362 × 0.3670) + (*m*/*z* 1525 × 0.2994) + (*m*/*z* 1566 × 0.2181) + (*m*/*z* 1591 × 0.3501) + (*m*/*z* 1607 × −0.0316) + (*m*/*z* 1648 × −0.4001) + (*m*/*z* 1687 × 0.2314) + (*m*/*z* 1709 × −1.9646) + (*m*/*z* 1753 × −1.3974) + (*m*/*z* 1769 × −0.1732) + (*m*/*z* 1794 × −0.4953) + (*m*/*z* 1810 × 1.6191) + (*m*/*z* 1849 × −1.0837) + (*m*/*z* 1871 × 0.1763) + (*m*/*z* 1915 × −0.0203) + (*m*/*z* 1956 × 0.7082) + (*m*/*z* 2011 × −0.6413) + (*m*/*z* 2033 × −0.6367) + (*m*/*z* 2058 × 0.9117) + (*m*/*z* 2074 × −0.6367) + (*m*/*z* 2220 × 0.9062) + (*m*/*z* 2337 × 0.9508) + (*m*/*z* 2379 × 1.0798) + (*m*/*z* 2525 × 0.2243) + (*m*/*z* 2728 × −1.4954) + (*m*/*z* 2744 × 1.0543) + (*m*/*z* 2890 × −1.1626) + (*m*/*z* 3049 × 0.4017) + (*m*/*z* 3109 × −0.5170) + (*m*/*z* 3195 × −0.3882) + (*m*/*z* 3341 × 0.1788) + (*m*/*z* 3414 × 0.6209) + (*m*/*z* 3560 × 0.1269) + (*m*/*z* 3719 × −1.1201) + (*m*/*z* 3865 × 0.4113) + (1.5761) (1)

The *N*-glycan score was calculated by multiplying the level of candidate objective variables by objective function values. The ABMR predictive performance of the *N*-glycan score was assessed by receiver operating characteristic (ROC) curve and Kaplan–Meier curve analyses.

### 2.2. The ABMR Predictive Performance of N-Glycan Score Based on the Aberrant Serum N-Glycan Profile Was Far Superior to That of Preformed Donor-Specific Antibody Status

**Figure 4:**
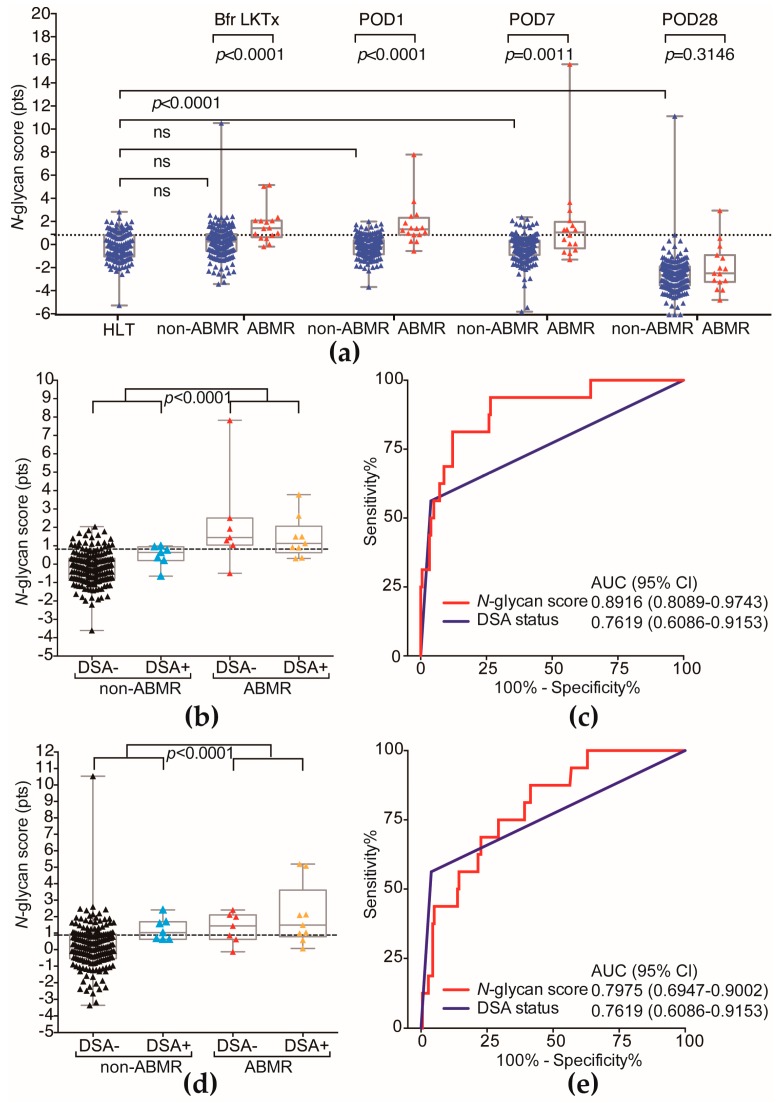
Prediction of ABMR by *N*-glycan score or preformed DSA. (**a**) Longitudinal follow-up of the *N*-glycan scores in the recipients who developed ABMR and those who did not. The *N*-glycan score of HLT was not significantly different from Bfr LKTx, postoperative day 1 (POD1) and POD7; (**b**) The *N*-glycan scores at POD1 in the recipients who developed ABMR and those who did not; (**c**) Receiver operating characteristic (ROC) curve analysis of *N*-glycan score at POD1 and preformed DSA status for detection of ABMR; (**d**) The level of the *N*-glycan score before LKTx (Bfr LKTx) in the recipients who developed ABMR and those who did not; (**e**) ROC curve analysis of the *N*-glycan score at Bfr LKTx and preformed DSA status for detection of ABMR.

The *N*-glycan scores one day before LKT (Bfr LKTx), at postoperative day 1 (POD1) and at POD7 were significantly higher in the recipients who developed ABMR (Figure 4a,b,d). The *N*-glycan score of HLT was not significantly different to the *N*-glycan score of non-ABMR groups at Bfr LKTX, at POD1 and POD7. Longitudinal follow-up at POD28 showed that there was no significant difference in the *N*-glycan score between the non-ABMR and ABMR groups. ROC curves were then used to compare the diagnostic performance between preformed DSA status and *N*-glycan score for ABMR prediction (Figure 4c,e). The area under the curve (AUC) of preformed DSA status and *N*-glycan score before LKTx and at POD1 for the prediction of ABMR were determined (preformed DSA AUC, 0.7619; *N*-glycan score before LKTx, 0.7975; *N*-glycan score at POD1, 0.8916). At the cutoff *N*-glycan score (0.8770 points) on POD1 for prediction of ABMR, the diagnostic accuracy was 86.29%, the positive predictive value was 81.25%, and the negative predictive value was 86.74%. The positive predictive value was much higher than that of preformed DSA (56.25%) (Table 4), which suggests that the *N*-glycan score can detect ABMR recipients with preformed DSA-negative status.

**Figure 5:**
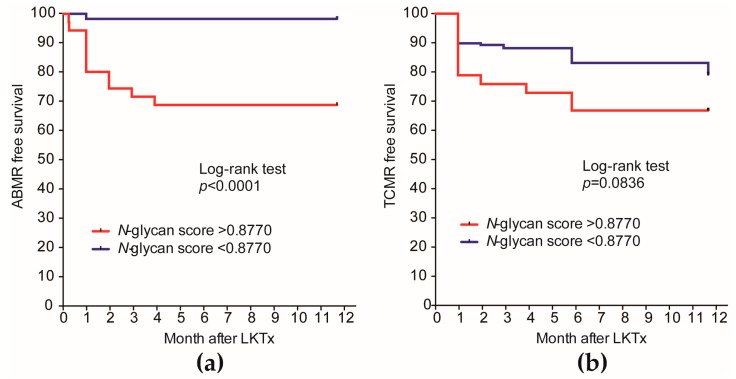
Kaplan–Meier curves for ABMR-free survival and TCMR-free survival of the recipients classified by *N*-glycan score status. (**a**) The recipients with *N*-glycan scores >0.8770 had significantly worse ABMR-free survival; (**b**) The recipients with *N*-glycan scores >0.8770 did not show a significant difference in TCMR-free survival.

Furthermore, the recipients with an *N*-glycan score greater than the cutoff value (0.8770) had significantly worse ABMR-free survival (log-rank test, *p* < 0.0001) (Figure 5a), but there was no significant difference in TCMR-free survival (log-rank test, *p* = 0.0836) (Figure 5b).

### 2.3. N-Glycan-Carrying Serum Immunoglobulin (Igs) Levels Were Not Significantly Different between the ABMR Group and Non-ABMR Group

**Figure 6:**
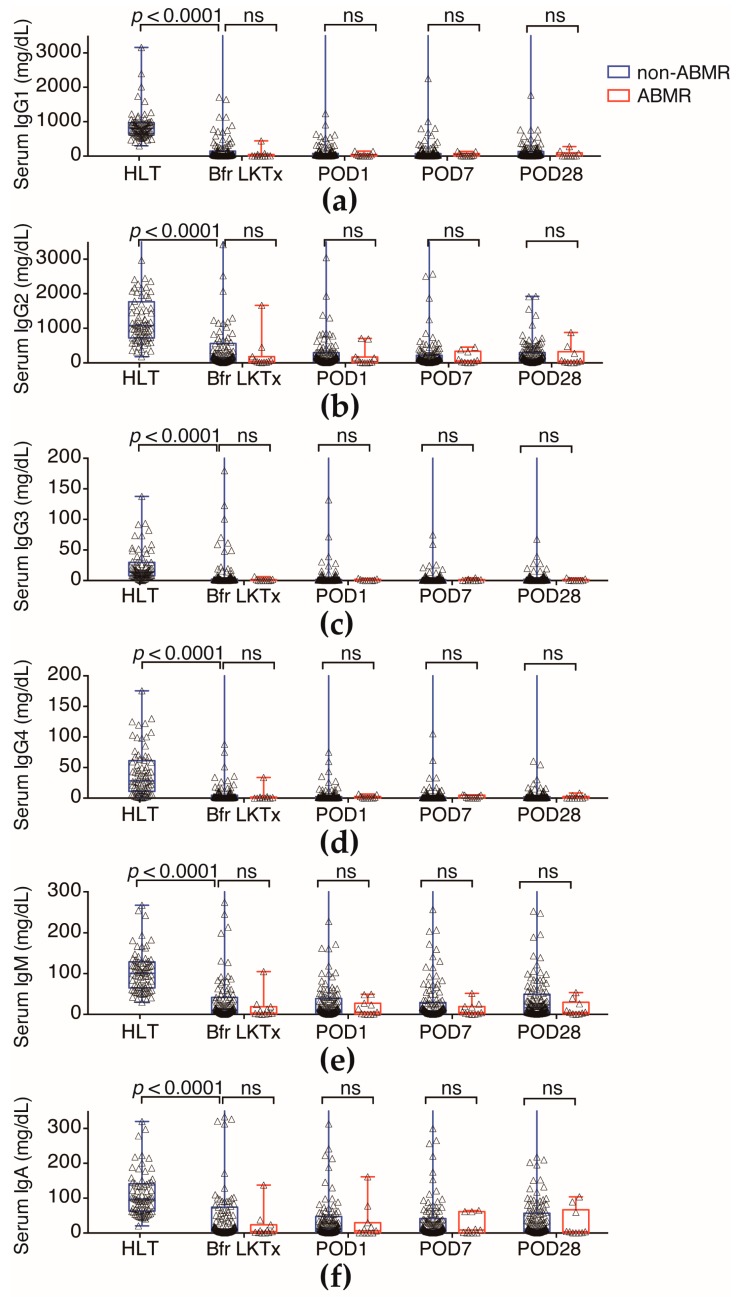
(**a**) Serum IgG1 levels in patients in HLT, non-ABMR, and ABMR patients; (**b**) Serum IgG2 levels in HLT, non-ABMR, and ABMR patients; (**c**) Serum IgG3 levels in HLT, non-ABMR, and ABMR patients; (**d**) Serum IgG4 levels in HLT, non-ABMR, and ABMR patients; (**e**) Serum IgM levels in HLT, non-ABMR, and ABMR patients; (**f**) Serum IgA levels in HLT, non-ABMR, and ABMR patients. All Igs levels in HLT were much higher than the LKTx group.

Serum *N*-glycomics revealed that the level of sialyl hybrid-type and sialyl bisecting-type *N*-glycans were significantly lower in the ABMR group. Serum *N*-glycomics may detect *N*-glycan-carrying glycoproteins in serum, such as Ig*s* (IgGs, IgA, and IgM)*,* which are major *N*-glycosylated proteins in serum [12]. Therefore, we analyzed serum Ig*s* levels in all samples. Although serum Ig*s* levels of all recipients were much lower than the benign level because of the administration of immunosuppressants, longitudinal follow-up of serum Igs levels before LKTx and on POD1, POD7, and POD28 after LKTx were not significantly different between the ABMR group and non-ABMR groups (Figure 6a–f).

**Figure 7:**
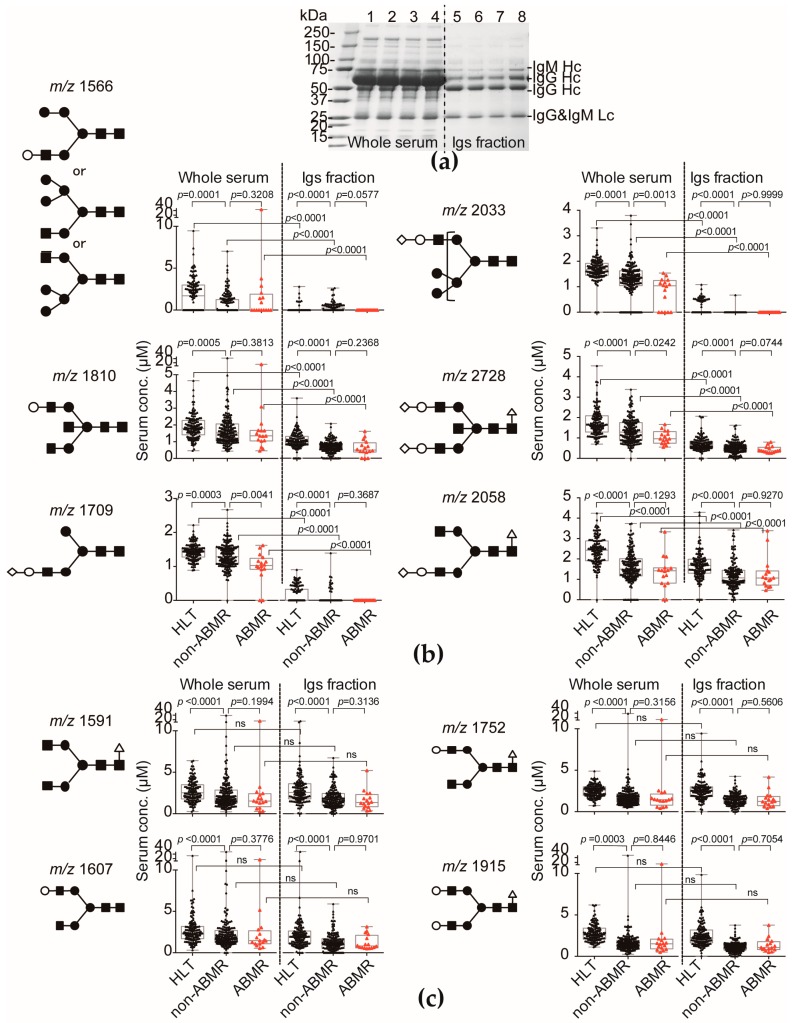
Levels of the ABMR-associated sialyl hybrid-type *N*-glycans in whole serum or in the immunoglobulin (Ig) fraction from patients before LKTx and on POD1. (**a**) CBB-stained band patterns on SDS-PAGE for representative whole-serum and purified Ig*s* fraction samples; (**b**) Levels of serum sialyl hybrid-type *N*-glycans (*m*/*z* 1566, 1709, and 2033) and bisecting-type *N*-glycans (*m*/*z* 1810, 2728, and 2058) in the whole-serum and Ig*s* fraction from non-ABMR and ABMR patients; (**c**) Levels of serum biantennary *N*-glycans (*m*/*z* 1591, 1607, 1753, and 1915) in the whole-serum and Ig*s* fraction from non-ABMR and ABMR patients. Putative structures of *N*-glycans are indicated by monosaccharide symbols.

To characterize the *N*-glycan profile of serum Ig*s*, non-Ig*s* proteins were eliminated by Melon Gel column chromatography (Figure 7a, lanes 5–8) and then compared with whole serum (lanes 1–4). *N*-glycomics of the Ig*s* fraction showed that the levels of *N*-glycans in the Ig*s* fraction were significantly lower than those in the whole-serum samples (Figure 7b) and Igs levels in LKTx patients were significantly lower than that of HLT. Although three ABMR-related terminal sialylated *N*-glycans (hybrid type: *m*/*z* 2033; bisecting type: *m*/*z* 2728; complex biantennary type: *m*/*z* 1709) in the whole serum levels were significantly lower in the recipients who developed ABMR than in those who did not, it is noteworthy that these three *N*-glycans in the Igs fraction was not significantly changed between non-ABMR and ABMR groups. Furthermore, these three *N*-glycans in the Igs fraction was significantly lower than in those of whole serum. The level of other ABMR-related sialylated *N*-glycans (bisecting-type: *m*/*z* 1810 and 2728; biantennary-type: *m*/*z* 2058) in the Ig*s* fraction was not significantly changed between non-ABMR and ABMR groups. The levels of biantennary *N*-glycans (*m*/*z* 1591, 1607, 1753, and 1915), which were not selected as ABMR-related *N*-glycans, were not significantly different between the Ig*s* fractions and whole-serum (Figure 7c). These results suggest that ABMR-related *N*-glycan change did not mainly originate from aberrant *N*-glycosylation of Igs.

## 3. Discussion

*N*-glycomics is a promising methodology, and several studies have shown that differences in glycan profiles between diseased and benign states may be useful in the diagnosis or prognosis of diseases [8,9,10,13,14]. In the present study, serum *N*-glycomics was used for recipients who developed ABMR within one month after LKTx. To the best of our knowledge, this is the first report to identify serum-aberrant *N*-glycosylation profiles as predictive biomarkers for ABMR in LKTx. Our results revealed that the *N*-glycan scores based on the whole aberrant *N*-glycosylation profile in serum one day before LKTx, at POD1, and at POD7 were significantly higher in the recipients who developed ABMR (Figure 4a,b,d). Longitudinal follow-up at POD28 showed that the *N*-glycan scores were not significantly different between the two groups. This finding suggests that the *N*-glycan score may reflect the early phase of the ABMR reaction in recipients who develop ABMR. Especially, we demonstrated that serum sialyl hybrid type and bisecting type *N*-glycans (*m*/*z* 1709, 2033, and 2728) on POD1 in the ABMR group were significantly lower than those in the non-ABMR group (Figure 3). One study showed a higher level of sialylated antibodies on the day of the transplant and at first DSA detection in patients who had good transplant outcomes [7]. Hess et al. reported that T-cell-independent B-cell activation was associated with the production of immunosuppressive sialylated serum IgGs, which inhibit B-cell activation and immune reactions, independent of FcγRIIB [15]. In addition, IgG molecules can perform pro- and anti-inflammatory effector functions depending on the composition of the fragment crystallizable (Fc) domain glycan. Quast et al. reported that IgG Fc sialylation of human monoclonal IgG1 molecules impaired their ability to induce complement-mediated cytotoxicity [16]. They also reported that the presence of sialic acid abrogated the increased binding of C1q to Fc-galactosylated IgG1 and resulted in decreased levels of C3b deposition on the cell surface [16]. Several reports have suggested that Fc-sialylated IgGs affect B-cell activation and complement-mediated cytotoxicity. Several previous reports and the present study results suggest that decreased amounts of sialylated *N*-glycans on serum glycoproteins may be associated with ABMR in LKTx. It remains unclear why ABMR-associated *N*-glycan downregulation on serum glycoprotein occurs and what kind of carrier proteins are involved in ABMR-associated changes in the serum *N*-glycan pattern. Ig*s* are major *N*-glycosylated proteins in serum [12]. Thus, we hypothesized that serum *N*-glycan profiles might reflect an *N*-glycosylation change of Ig*s* in ABMR patients. However, in our study, total Igs levels of LKTx patients were significantly lower than healthy people and ABMR-associated *N*-glycans (*m*/*z* 1566, 1709 and 2033) were not detected in Ig*s* fractions (Figure 6b). In contrast, concentrations of biantennary *N*-glycans (*m*/*z* 1591, 1607, 1752, and 1915) did not differ significantly between the Ig*s* fractions and whole serum and between the ABMR and non-ABMR groups (Figure 6c). These findings suggest that the major carrier protein(s) of ABMR-related sialyl hybrid-type *N*-glycans do not originate from Ig*s*. This result was not reconciled in the previous study of Malard-Castagnet et al. They focus on higher levels of terminal sialylated IgG in DSA-positive patients which were detectable on the day of the transplant in those who did not develop ABMR, and also did not identify which sialylated *N*- or *O*-glycan structure on IgG was associated with ABMR. In this study, we focused our comprehensive *N*-glycan analysis of whole serum and Igs fractions (mixture of IgG, IgM, and IgA) in both DSA-positive and DSA-negative patients who developed ABMR or not. The patient background and method of our study is completely different from Malard-Castagnet et al. Thus, we hypothesized that both the reduced sialylation of IgG and whole aberrant *N*-glycan profile changes of serum glycoproteins, except for Igs, may occur in recipients who develop ABMR.

The other possibility is that the serum levels of free *N-*glycans change in ABMR. Recently, Seino et al. demonstrated that levels of disialylated free *N*-glycans in serum samples were higher in patients with hepatocellular carcinoma than in healthy controls [17]. Nonetheless, their results showed 100-fold lower amounts of disialylated free *N*-glycans in serum than those shown by our glycoblotting method, and their free *N*-glycan analysis did not detect sialyl hybrid-type *N*-glycans in serum samples. This observation suggests that our high-throughput *N*-glycomics detects not only free *N*-glycans in serum, but also *N*-glycans derived from serum glycoproteins.

Another possible carrier serum protein is α-1-acid glycoprotein (AGP), which is secreted from the liver into plasma [18]. AGP has been studied as an acute-phase serum glycoprotein that possesses five *N*-linked complex type heteroglycan side chains, which may be present as biantennary, triantennary, or tetra-antennary structures. Additionally, AGP has been studied in association with inflammation, autoimmune diseases, and cancer [19]. Although the origin and clinical implications of serum *N*-glycans remain unclear, our ongoing studies address these issues and show potential clinical utility. Several previous reports and the present study results suggest that decreased amounts of sialyl hybrid-type *N*-glycans on serum glycoproteins, except for Ig*s*, may be associated with ABMR in LKTx. These results suggest that the use of *N*-glycomics may provide insights into new factors predicting ABMR.

We also demonstrated that the positive predictive value of the *N*-glycan score (81.25%) for detection of ABMR was significantly higher than that of preformed DSA status (56.25%). Although, preformed DSA was a powerful indicator of recipients who did not develop ABMR, some with preformed DSA-negative recipients developed ABMR. In the present study, we demonstrated that the *N*-glycan score could identify preformed DSA-negative recipients who developed ABMR. Thus, the *N*-glycan score may be a complement to preformed DSA status.

This was a small study in only 16 ABMR recipients, so the findings should be considered preliminary. To validate the proposed predictive biomarker of ABMR, a study with a greater number of patients is required. Despite this sample limitation, the results suggest that the serum aberrant *N*-glycan profile can reflect a systemic immunogenic reaction in the early ABMR state. Future studies should determine whether these alterations are a direct result of antibody-mediated allograft injury in LKTx recipients.

## 4. Materials and Methods

### 4.1. Ethics Statement

This study was performed in accordance with the ethical standards of the Declaration of Helsinki and was approved by the Ethics Committees of all participating institutions (Akita University Hospital, St. Marianna University of Medicine, Tokyo-Woman’s Medical University, Sapporo City General Hospital, and Hirosaki University Hospital) (“The study about carbohydrate structure change in urological disease”; approval number: 2014-195, 22 December 2014). Informed consent was obtained from all patients.

### 4.2. Serum Samples and Diagnosis of ABMR

A total of 753 recipients underwent LKTx at Akita University Hospital, St. Marianna University of Medicine, Tokyo-Woman’s Medical University, Sapporo City General Hospital, or Hirosaki University Hospital between 2007 and 2016. Of those serum available 197 recipients underwent LKTx were retrospectively selected from our serum bank. Healthy controls (HLT, *n* = 135) selected from community-dwelling volunteers in the health maintenance programme of Iwaki Health Promotion Project. Serum samples were collected one day before LKTx and on POD1, POD7, and POD28 and stored at −80 °C until use. Of those 197, 16 recipients with biopsy-proven ABMR with or without TCMR, 40 recipients with biopsy-proven TCMR, 141 patients without any adverse events, and 135 healthy controls were subjected to serum *N*-glycomic analysis using the glycoblotting method and matrix-assisted laser desorption ionization-time-of-flight mass spectrometry (MALDI-TOF MS) analysis. Clinical ABMR and TCMR were diagnosed according to Banff classifications by protocol and/or episode biopsy when we observed exacerbation of renal function [20]. Preformed DSA detection was evaluated by using a FlowPRA Single antigen kit (Veritas Corp., Tokyo, Japan) before LKTx. We also examined DSA by using a FlowPRA Single antigen kit when we found exacerbation of renal function and/or pathological abnormality by protocol or episode biopsy after LKTx.

### 4.3. Glycoblotting Method and Mass Spectrometry

**Figure 8:**
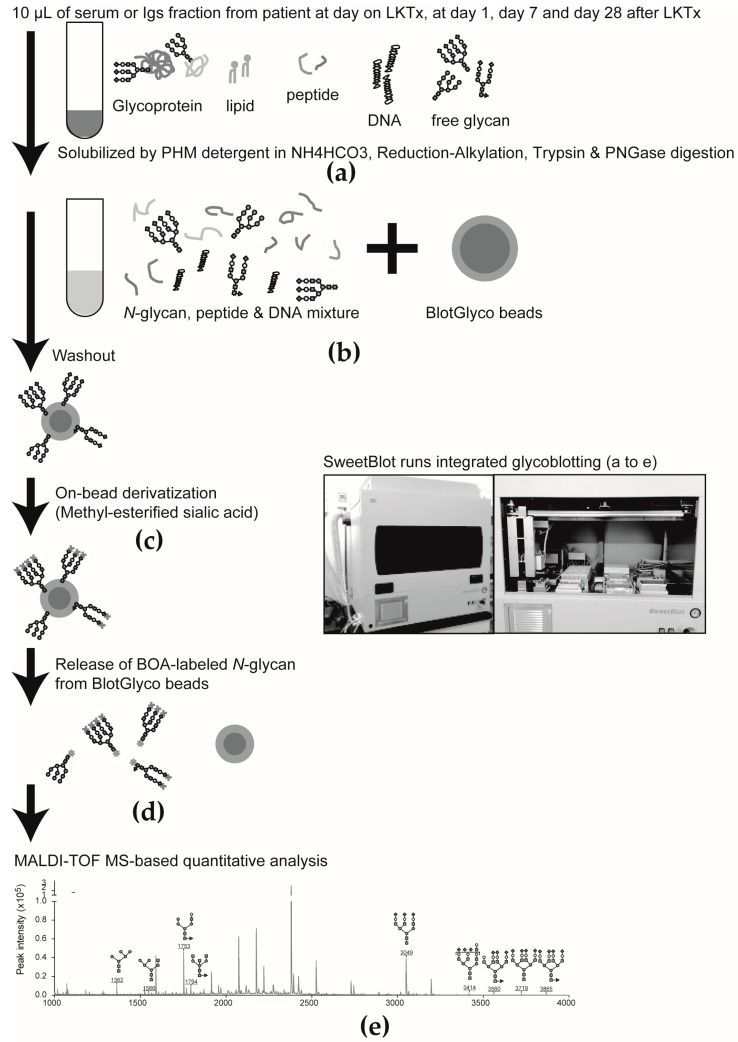
The general protocol for the integrated glycoblotting technique and workflow for glycoblotting-based high-throughput clinical glycan analysis. (**a**) Ten-microliter serum samples are applied to SweetBlot^TM^ for glycoblotting; (**b**) After enzymatic cleavage from serum protein, total serum *N-*glycans released into the digestion mixture were directly mixed with BlotGlyco H beads to capture *N-*glycans; (**c**) After the beads are separated from other molecules by washing, sialic acid is methyl esterified; (**d**) These processed *N-*glycans are then labeled with BOA and released from BlotGlyco H beads; (**e**) Mass spectra of BOA-labeled *N-*glycans are acquired by using an Ultraflex III instrument.

Serum *N*-glycomics was performed as described previously. A 10-μL aliquot of whole serum or a Igs fraction purified from whole serum was processed by using the glycoblotting method [8,9,10,11,12,13,14,21] and a controlled automated SweetblotTM instrument (System Instruments, Hachioji, Japan). Then, the resulting BOA-labeled glycans were detected by MALDI-TOF MS (Ultraflex 3 TOF/TOF mass spectrometer; Bruker Daltonics, Bremen, Germany) (Figure 8). Composition and structures of the glycans were predicted by using the GlycoMod Tool (http://br.expasy.org/tools/glcomod). Each quantitative reproducibility test of SweetBlot was performed as described elsewhere [22]. Quantitative reliability was then evaluated on the basis of the following parameters: outliers were allowed <3 points, slope of <3.0, and the significance level of the correlation coefficient *r* was <0.05. Glycan peaks were assumed to be useful when the above-mentioned criteria of the assay were met, and the resulting glycans were used for statistical analysis.

### 4.4. Statistical Analysis

All calculations for clinical data were performed by using SPSS software, ver. 21.0 (SPSS Inc., Chicago, IL, USA) and GraphPad Prism 6.03 (GraphPad Software, San Diego, CA, USA). Intergroup differences were statistically analyzed by performing Student’s *t* test for normally-distributed variables or by performing the Mann–Whitney *U* test for non-normally distributed models. Differences with *p* < 0.05 were considered to be significant. Multivariate discriminant analysis for prediction of ABMR was performed by inputting an ABMR event as an explanatory variable and sex, age, and *N*-glycans level as objective variables. The ABMR predictive *N*-glycan score was calculated by multiplying objective variables by objective function values. The ABMR predictive performance of the *N*-glycan scoring method was evaluated by ROC curve analysis. ROC curves were developed by using the library “rms” in R (http://www.r-project.org/) [23], and statistical differences between AUCs were calculated by using the same program. ABMR-free and TCMR-free survivals were evaluated by using Kaplan–Meier curves, and differences between groups were assessed by performing the log-rank test. Differences with *p* < 0.05 were considered to be significant.

## 5. Conclusions

The *N*-glycan score, based on the whole aberrant *N*-glycosylation profile, including downregulation of sialylated *N*-glycans in serum glycoprotein, was shown to be a promising predictive biomarker of early ABMR in this study. Future large-scale prospective validation studies may definitively determine the clinical utility of these carbohydrate biomarkers for ABMR prediction.

## Figures and Tables

**Table 1:** Thirty-six types of *N*-glycans that showed good quantitative reproducibility in all samples and could be analyzed statistically.

No.	*m*/*z*	Composition
1	1362.5	(Hex)_2_ + (Man)_3_(GlcNAc)_2_
2	1524.5	(Hex)_3_ + (Man)_3_(GlcNAc)_2_
3	1565.5	(Hex)_5_ + (HexNAc)_3_
4	1590.6	(HexNAc)_2_(dHex)_1_ + (Man)_3_(GlcNAc)_2_
5	1606.6	(Hex)_1_(HexNAc)_2_ + (Man)_3_(GlcNAc)_2_
6	1647.6	(HexNAc)_3_ + (Man)_3_(GlcNAc)_2_
7	1686.6	(Hex)_4_ + (Man)_3_(GlcNAc)_2_
8	1708.6	(Hex)_1_(HexNAc)_1_(NeuAc)_1_ + (Man)_3_(GlcNAc)_2_
9	1752.6	(Hex)_1_(HexNAc)_2_(dHex)_1_ + (Man)_3_(GlcNAc)_2_
10	1768.6	(Hex)_2_(HexNAc)_2_ + (Man)_3_(GlcNAc)_2_
11	1793.7	(HexNAc)_3_(dHex)_1_ + (Man)_3_(GlcNAc)_2_
12	1809.7	(Hex)_1_(HexNAc)_3_ + (Man)_3_(GlcNAc)_2_
13	1848.6	(Hex)_5_ + (Man)_3_(GlcNAc)_2_
14	1870.7	(Hex)_2_(HexNAc)_1_(NeuAc)_1_ + (Man)_3_(GlcNAc)_2_
15	1914.7	(Hex)_2_(HexNAc)_2_(dHex)_1_ + (Man)_3_(GlcNAc)_2_
16	1955.7	(Hex)_1_(HexNAc)_3_(dHex)_1_ + (Man)_3_(GlcNAc)_2_
17	2010.7	(Hex)_6_ + (Man)_3_(GlcNAc)_2_
18	2032.7	(Hex)_3_(HexNac)_1_(NeuAc)_1_ + (Man)_3_(GlcNAc)_2_
19	2057.8	(Hex)_1_(HexNAc)_2_(dHex)_1_(NeuAc)_1_ + (Man)_3_(GlcNAc)_2_
20	2073.8	(Hex)_2_(HexNAc)_2_(NeuAc)_1_ + (Man)_3_(GlcNAc)_2_
21	2219.8	(Hex)_2_(HexNAc)_2_(dHex)_1_(NeuAc)_1_ + (Man)_3_(GlcNAc)_2_
22	2336.9	(Hex)_3_(HexNAc)_4_ + (Man)_3_(GlcNAc)_2_
23	2348.9 ^1^	Internal standard (BOA-labeled A2 amide)
24	2378.9	(Hex)_2_(HexNAc)_2_(NeuAc)_2_ + (Man)_3_(GlcNAc)_2_
25	2524.9	(Hex)_2_(HexNAc)_2_(dHex)_1_(NeuAc)_2_ + (Man)_3_(GlcNAc)_2_
26	2727.9	(Hex)_2_(HexNAc)_3_(dHex)_1_(NeuAc)_2_ + (Man)_3_(GlcNAc)_2_
27	2743.9	(Hex)_3_(HexNAc)_3_(NeuAc)_2_ + (Man)_3_(GlcNAc)_2_
28	2890.1	(Hex)_3_(HexNAc)_3_(dHex)_1_(NeuAc)_2_ + (Man)_3_(GlcNAc)_2_
29	3049.1	(Hex)_3_(HexNAc)_3_(NeuAc)_3_ + (Man)_3_(GlcNAc)_2_
30	3109.1	(Hex)_4_(HexNAc)_4_(NeuAc)_2_ + (Man)_3_(GlcNAc)_2_
31	3195.2	(Hex)_3_(HexNAc)_3_(dHex)_1_(NeuAc)_3_ + (Man)_3_(GlcNAc)_2_
32	3341.2	(Hex)_3_ (HexNAc)_3_ (Deoxyhexose)_2_ (NeuAc)_3_ + (Man)_3_(GlcNAc)_2_
33	3414.2	(Hex)_4_(HexNAc)_4_(NeuAc)_3_ + (Man)_3_(GlcNAc)_2_
34	3560.3	(Hex)_4_(HexNAc)_4_(dHex)_1_(NeuAc)_3_ + (Man)_3_(GlcNAc)_2_
35	3719.3	(Hex)_4_(HexNAc)_4_(NeuAc)_4_ + (Man)_3_(GlcNAc)_2_
36	3865.4	(Hex)_4_(HexNAc)_4_(dHex)_1_(NeuAc)_4_ + (Man)_3_(GlcNAc)_2_

^1^ *m*/*z* 2348.9 is the internal standard, disialo-galactosylated biantennary *N*-glycan, which contains amidated sialic acid residues (A2 amide glycans). Compositional annotations and putative structures are shown as the following abbreviations. Hex: hexose; HexNAc: *N*-acetylhexosamine; dHex: deoxyhexose.

**Table 2:** Demographics of the patients in each cohort.

	HLT ^5^	All LKTx ^2^ Cases	Non-ABMR ^a^		ABMR ^b^	*p* Value (^a^ vs. ^b^)
			No Event	TCMR	ABMR ± TCMR	
Number of recipients	135	197	141	40	16	
Recipient’s sex (male/female)	93/42	108/89	76/65	26/14	6/10	ns ^1^
Recipient’s age at LKTx ^2^ (median, IQR ^3^)	38 (24–67)	52 (40–59)	52 (40–59)	51 (39–58)	51 (43–58)	ns ^1^
ABOi KTx ^4^		97 (49%)	63 (44%)	24 (60%)	10 (62.5%)	ns ^1^
Rituximab		79 (40%)	50 (36%)	17 (43%)	12 (75%)	0.0003
Preformed DSA-positive status		16 (8.1%)	6 (4%)	1 (3%)	9 (56%)	0.0003
Non-ABMR survival, month (range)		48.5 (0.001–117.9)	40.8 (7.7–117.9)	75.1 (16.2–114.7)	1 (0.001–4)	<0.0001
Non-TCMR survival, month (range)		24.1 (0.25–117.9)	40.4 (7.7–117.9)	1 (1–12)	8.8 (0.25–96.3)	0.0039

^1^ Not significant; ^2^ living kidney transplant; ^3^ interquartile range; ^4^ ABO incompatible kidney transplant; ^5^ Healthy volunteer. ^a^ Non-antibody mediated rejection include No event and TCMR group; ^b^ Antibody mediated rejection include ABMR ± TCMR group.

**Table 3:** Multivariate discriminant analysis for prediction of ABMR.

Variables	Wilk’s Lambda	*F* Value	ODF ^1^	TDF ^2^	*p* Value	Function Value
Recipient sex	0.9735	4.3294	1	159	0.0391	−0.9142
Recipient Age at LKTx ^3^	0.9999	0.0201	1	159	0.8873	−0.0024
*m*/*z* 1362	0.9992	0.1254	1	159	0.7238	0.3670
*m*/*z* 1525	0.9997	0.0457	1	159	0.8311	0.2994
***m*/*z* 1566**	**0.9821**	**2.8998**	**1**	**159**	**0.0905**	**0.2181**
*m*/*z* 1591	0.9969	0.4931	1	159	0.4836	0.3501
*m*/*z* 1607	0.9999	0.0224	1	159	0.8811	−0.0316
*m*/*z* 1648	0.9938	0.9880	1	159	0.3217	−0.4001
*m*/*z* 1687	0.9996	0.0581	1	159	0.8098	0.2314
***m*/*z* 1709**	**0.9857**	**2.3116**	**1**	**159**	**0.1304**	**−1.9646**
*m*/*z* 1753	0.9882	1.8956	1	159	0.1705	−1.3974
*m*/*z* 1769	0.9966	0.5473	1	159	0.4605	−0.1732
*m*/*z* 1794	0.9969	0.5001	1	159	0.4805	−0.4953
***m*/*z* 1810**	**0.9817**	**2.9575**	**1**	**159**	**0.0874**	**1.6191**
*m*/*z* 1849	0.9928	1.1510	1	159	0.2850	−1.0837
*m*/*z* 1871	0.9995	0.0823	1	159	0.7746	0.1763
*m*/*z* 1915	1.0000	0.0005	1	159	0.9818	−0.0203
*m*/*z* 1956	0.9967	0.5323	1	159	0.4667	0.7082
*m*/*z* 2011	0.9962	0.6075	1	159	0.4369	−0.6413
***m*/*z* 2033**	**0.9860**	**2.2501**	**1**	**159**	**0.1356**	**−0.6367**
*m*/*z* 2058	0.9870	2.1008	1	159	0.1492	0.9117
*m*/*z* 2074	0.9999	0.0216	1	159	0.8834	0.2810
*m*/*z* 2220	0.9975	0.3938	1	159	0.5312	0.9062
*m*/*z* 2337	0.9992	0.1319	1	159	0.7169	0.9508
*m*/*z* 2379	0.9991	0.1483	1	159	0.7006	1.0798
*m*/*z* 2525	0.9998	0.0373	1	159	0.8472	0.2243
***m*/*z* 2728**	**0.9846**	**2.4851**	**1**	**159**	**0.1169**	**−1.4954**
*m*/*z* 2744	0.9989	0.1678	1	159	0.6826	1.0543
*m*/*z* 2890	0.9963	0.5937	1	159	0.4421	−1.1626
*m*/*z* 3049	0.9998	0.0383	1	159	0.8451	0.4017
*m*/*z* 3109	0.9938	0.9901	1	159	0.3212	−0.5170
*m*/*z* 3195	0.9996	0.0689	1	159	0.7933	−0.3882
*m*/*z* 3341	0.9956	0.7013	1	159	0.4036	0.1788
*m*/*z* 3414	0.9986	0.2210	1	159	0.6389	0.6209
*m*/*z* 3560	0.9991	0.1355	1	159	0.7133	0.1269
*m*/*z* 3719	0.9904	1.5426	1	159	0.2161	−1.1201
*m*/*z* 3865	0.9962	0.6102	1	159	0.4359	0.4113
				constant term		1.5761

^1^ One degree of freedom; ^2^ two degrees of freedom; ^3^ living kidney transplant. Bold values indicated that six *N*-glycans related to detection of ABMR that had calculated *F* values > 2.0 by multivariate discriminant analysis.

**Table 4:** Predictive value of *N*-glycan score at the cutoff point.

	**Cutoff Value of *N*-glycan Score**			
Diagnostic result	<0.8770	≥0.8770	Predictive value (%)	
Non-ABMR	157	24	86.74	NPV ^1^
ABMR	3	13	81.25	PPV ^2^
Total	160	37	86.29	Accuracy
	**Preformed DSA status**			
Diagnostic result	Negative	Positive	Predictive value (%)	
Non-ABMR	174	7	96.13	NPV ^1^
ABMR	7	9	56.25	PPV ^2^
Total	160	16	92.89	Accuracy

^1^ Negative predictive value; ^2^ positive predictive value.

## Abbreviations

ABMR	Antibody-mediated rejection
LKTx	Living donor kidney transplant
TCMR	T-cell-mediated rejection
ROC	Receiver operating characteristic
AUC	Area under the curve
DSA	Donor-specific anti-HLA antibodies
IgG	Immunoglobulin G
Gal	Galactose
Man	Mannose
GlcNAc	*N*-acetylglucosamine
Fuc	Fucose
Sia	Sialic acid
Hex	Hexose
HexNAc	*N*-acetylhexosamine
dHex	Deoxyhexose
MALDI-TOF MS	Matrix-assisted laser desorption ionization-time-of-flight mass spectrometry
BOA	Benzyloxyamine
ABOi	ABO incompatible
KTx	Kidney transplant
ODF	One degree of freedom
TDF	Two degrees of freedom
Bfr	Before
POD	Postoperative day
PRA	Panel reactive antibody
Igs	Immunoglobulins
